# Scan path entropy and arrow plots: capturing scanning behavior of multiple observers

**DOI:** 10.3389/fpsyg.2013.00996

**Published:** 2013-12-24

**Authors:** Ignace Hooge, Guido Camps

**Affiliations:** Department of Experimental Psychology, Faculty of Social Sciences, Helmholtz Institute, Utrecht UniversityUtrecht, Netherlands

**Keywords:** scan-path, entropy, eye-tracking, gaze-guidance, data visualization

## Abstract

Designers of visual communication material want their material to attract and retain attention. In marketing research, heat maps, dwell time, and time to AOI first hit are often used as evaluation parameters. Here we present two additional measures (1) “scan path entropy” to quantify gaze guidance and (2) the “arrow plot” to visualize the average scan path. Both are based on string representations of scan paths. The latter also incorporates transition matrices and time required for 50% of the observers to first hit AOIs (T_50_). The new measures were tested in an eye tracking study (48 observers, 39 advertisements). Scan path entropy is a sensible measure for gaze guidance and the new visualization method reveals aspects of the average scan path and gives a better indication in what order global scanning takes place.

## Introduction

Marketing research companies use eye trackers to give layout advice to enhance advertisements (Pieters and Wedel, 2004; Pieters et al., 2007). This can be done, for example, by comparing gaze behavior on (slightly) different designs. A number of observers look at different designs, and based on how fast a certain element [e.g., brand label, visual or text message (Pieters and Wedel, 2004)] is fixated for the first time, the best design can be chosen. Based on the obtained eye movements advice can be given to slightly change the design. Examples of such advice are making a logo more conspicuous or removing a distracter that hampers message transfer. Heat maps (also referred to as attention maps) are often used to visualize the eye movement data in this evaluation process. Despite the benefits of heat maps to visualize eye movements, it is desirable to have a method to show more of the average scanning behavior of the whole population of observers. Is it possible to visualize the average scan path? This seems a simple question, but scan paths have both temporal and spatial properties and may differ a lot between individual observers. We are convinced that layout advice would gain from more sophisticated eye tracking methods than those that are available nowadays. In this study we suggest two quantitative measures combined with a visualization method to reveal properties of average scanning behavior. The visualization method is based on transition matrices (Goldberg and Kotval, 1999) and a visualization method proposed by Lessing and Linge (2002). The timing measure is based on T_50_, a reaction time measure that takes into account that areas of interest (from now on referred to as AOI) are not always fixated by all observers in the test (Montfoort et al., 2007). The measure for the spatial aspects of scanning, scan path entropy, is new.

The goal of visual communication material (e.g., ads, road signs, warnings) is to transfer a message effectively. “Message” should be interpreted here in its broadest sense; it may be a literal text message like “Drink Coke,” but it may also the message that parking on the left side of the road is prohibited. The first requisite for visual message transfer is that people perceive the message. Perception may be a problem because the resolution of the retina decreases with eccentricity. A visual stimulus in the periphery may be too small to be resolved by the peripheral retina. There is a second problem for perception of elements in the periphery. Especially in cluttered visual scenes crowding may hamper perception. Crowding is the phenomenon that elements that look like a target element (a brand logo) laterally mask the target (Bouma, 1970; Toet and Levi, 1992; Vlaskamp and Hooge, 2006). The terminology may differ but in the marketing literature effects of visual context have been investigated (Pieters et al., 2007). The negative effects on perception of both the lower resolution of the peripheral retina and crowding can be reduced by making saccadic eye movements. By means of a saccade interesting elements are projected onto the fovea (the most sensitive area of the retina), to make sampling at the highest resolution possible. Another important factor in collecting visual information is time. Fixation time (time between two succeeding saccades) has to be long enough to enable encoding of the visual information around the fixation point. If fixation time is too short to allow for visual encoding, observers may re-fixate (Hooge and Erkelens, 1996; Hooge et al., 2007). There are of course many other factors that play an important role in message transfer (memory, state of mind, language, culture etc.). However, in the chain of collecting visual information, fixation is the first step. Without fixation, in most cases perception is impossible or hampered and the succeeding processes have no chance to succeed.

On top of the previous, in the real world message transfer suffers from additional factors. Concurrent messages compete for attention and exposure time is often limited. Imagine that you drive your car with 120 km/h and a company tries to reach you by means of a billboard standing along the road among other billboards. Similarly, static messages in television commercials are presented for a limited amount of time. The previous sets the prerequisites for ads; the more effective ad is the ad that that has the capability to transfer its message more quickly.

Eye tracking is a logical step in testing and improving visual material because fixations play an important role in gathering visual information. The usual way to investigate gaze behavior is by means of AOIs. Usually AOIs are drawn by hand and commercial packages (Tobii Studio, BeGaze from SMI and Data viewer from EyeLink) have the ability to compute most AOI measures such as dwell time, transition rate and time to first hit (Holmqvist et al. chapter 6). Dwell time and total dwell time are good measures for attention retention capacity; time to first hit is useful to estimate attention-capturing capacity of a visual element.

In manmade images such as paintings, drawings and ads the elements are often organized spatially. For example, ads are designed according to a traditional composition with brand logo in the right bottom corner acting like a metaphorical sender. One may expect that traditional composition may help to increase the number of fixations at the logo. However, we do not know whether this is true. In other cases designers may have a hypothesis or an intention with their design to guide gaze to a certain location. A good example of clever design can be found in the work of Oliviero Toscani who designed many controversial Benetton ads. The “Food for Life” ad of 1997 and the “White, Black and Yellow” ad seem to have gaze-guiding properties. Humans seem to be good eye trackers themselves, it is well known that gaze direction of one person is a strong cue for another person to direct attention (and gaze) to the gazed at location (Frischen et al., 2007). There are also attempts to actively guide gaze to certain locations by subtle visual cues (Barth et al., 2006a,b; McNamara et al., 2008). Magicians point with their hands and use their gaze and beautiful assistants to distract the audiences gaze from the actual locations where they do the trick. Here we will refer to gaze guidance if a visual stimulus has the (implicit and sometimes unintentional) capability to bias gaze systematically in a certain direction to increase the number of observers fixating a specific element such as the brand logo or to decrease time to brand logo fixation by guiding the eyes directly to it. Here we hypothesize that visual stimuli with good gaze guiding capacities produce similar scan paths in different observers.

Gaze guidance in relation to whole scan paths is a new topic, however, there is a long history of scan path research and this field is still very active (Noton and Stark, 1971; Brandt and Stark, 1997; Goldberg and Kotval, 1999; Cristino et al., 2010; Jarodzka et al., 2010; Dewhurst et al., 2012; Mathot et al., 2012). This literature focuses on scan path of individuals and methods to compare individual scan paths. In contrast, we are interested in describing and visualizing scan paths of a population of observers to investigate gaze guidance in visual stimuli. Such description will be useful in marketing research, investigation of art but also in the psychology of scene perception and visual search. What are the minimal requirements for such a description? The measures and visualizations should be capable of capturing temporal order, and spatial layout properties of scan paths of a population of observers.

As stated before, scan paths of individual observers should resemble each other more if gaze guidance is present and effective. A first attempt to investigate the effectiveness of gaze guidance is done by studying all scan paths of a group of observers at the same visual stimulus. We coded and subsequently counted the scan paths to produce a scan path histogram. Here we sketch two possible extremes: all observers produce different scan paths, the other extreme is that all observers follow a similar scan path, which can be seen as ordered group behavior. Information theory has a measure to describe the information in a variable in terms of ordering. This measure is Shannon entropy and it is defined as:

(1) $$H\left(X\right)=-\sum_{i=1}^{n}p{\left(x_{i}\right)}^{2}\log\;p\left(x_{i}\right)$$

where *H*(*X*) is the entropy in bits and *p*(*x_i_*) is the proportion of measurement *x_i_*. The idea behind entropy is as follows. If we throw a die, it has 6 possible outcomes (*x* = 1, *x* = 2, *x* = 3, *x* = 4, *x* = 5, and *x* = 6) and the chance on each of these outcomes is 1/6. We can apply the entropy formula (which means adding up the information values and weigh them with their chance of occurrence), resulting in *H*(*X*) = 2.5850 bits. Imagine that the die is loaded [the new manipulated chances are: *P*(x = 1) = 0.1, *P*(x = 2) = 0.1, *P*(x = 3) = 0.1, *P*(x = 4) = 0.1, *P*(x = 5) = 0.1 and *P*(x = 6) = 0.5]. Now entropy becomes lower, formula (1) gives *H*(X) = 2.161 bits. A loaded die is a metaphor for a visual stimulus biasing scan behavior. Biased scan behavior results in lower scan path entropy. In this study we measure eye movements in different advertisements and investigate whether scan path entropy is a sensible measure. If we succeed, we will have a measure (one number) to describe scanning behavior of a group of observers. In other words, scan path entropy can be used to quantify gaze guiding properties of a visual stimulus.

Temporal aspects of scanning are at least as interesting as spatial ones. Even if different observers follow similar scan paths, their behavior may differ a lot because some people fixate long, where others have a much higher saccade rate. For example people are known to fixate longer with increasing age (Spooner et al., 1980; Abel et al., 1983). To determine attention attraction power of an area of interest we could simply compute average time to first AOI hit. However average reaction time will not provide us with this information in certain situations and that needs some explanation. Imagine we engage 100 observers in a fictitious experiment with one visual stimulus yielding two AOIs. The observers were asked to watch the stimulus for 1.5 s. The data revealed that 45 unique observers fixated AOI nr 1 for the first time after on average 521 ms. AOI nr 2 was fixated by 21 unique observers and their average time to AOI first hit was 309 ms. Based on these two measures we cannot decide which of the two AOIs has the highest attention attraction power. AOI nr 1 attracts a higher number of fixations than AOI nr 2, but needs more time to achieve that. AOI nr 2 quickly receives a lot of attention, but is not very successful in attraction a lot of attention. This problem can be solved with a measure presented in Montfoort et al. (2007). They used a measure called T_0.5_ instead of averaged RT to enable comparison of reaction times produced by two groups (here we refer to T_50_ instead of T_0.5_). In their experiment one group has high accuracy and long reaction times, the other group had low accuracy and shorter reaction time.

Now we have measures for gaze guidance and attention attraction capacities. We all know the saying “a picture is worth a thousand words”, we need visualization that is more sophisticated than an attention map. A good visualization may help to explain test results to both customers of marketing research companies and designers in a more intuitive manner. The attention map is often used to illustrate gaze behavior of a group of observers, however, attention maps have many disadvantages. One disadvantage is that attention maps lack temporal ordering (Holmqvist et al., 2011, chapter 7 for a critical view on attention maps). Here we like to suggest a more sophisticated visualization based on the work of Lessing and Linge (2002). Linge and Lessing visualized a transition matrix (Stark and Ellis, 1981; Goldberg and Kotval, 1999) by means of arrows and numbers representing transitions from one AOI to another AOI and plotted those superimposed on the visual stimulus. They write “When the analysis is first presented there are no arrows, as arrows from every object to every other object can clutter the screen.” Their solution to solve this problem is elegant, they produce a figure called “Stand-alone diagram” with AOIs represented by circles with arrows and numbers between the AOIs to represent transition probabilities. But, we still prefer the original visualization because the visual stimulus is involved. Therefore we stick to the original Lessing and Linge (2002) approach but modified it and combined it with the timing measure from the previous section to add temporal order information. The modification consists of splitting up the visualization in two parts: one component describing eye movement traffic density and the other describing net eye movement direction.

Summarized, to facilitate evaluation of eye movements of a group observers while viewing a visual stimulus we

1. Introduce a new measure, scan path entropy, describing the distribution of scan paths of a group of observers and indicative of attention or gaze guiding capacity of the image
2. Introduce a better measure for saccadic reaction times to AOIs, T_50_ that stands for attention drawing capacity of an image element or AOI. This measure, when added to a spatial visualization, adds necessary temporal order information.
3. Present a visualization method that makes visual scanning behavior of a group more transparent than previous methods.

To test the quality of our new measures scan path entropy and T_50_, we engaged 54 subjects in an experiment with 70 different ads downloaded from the Internet and compared Scan Path Entropy and T_50_. To be sure that the results are not caused by a specific method of AOI production we, we produce our AOIs in two ways: (1) gridded AOIs, each ad is divided in 12 equally sized rectangles, (2) hand drawn AOIs of fixation clusters based on the heat map. We expect to find a high correlation between Scan Path Entropy and T_50_ irrespective of AOI production method. Of course a validation against another method would be preferable, but we have no knowledge of another method to quantify gaze guidance. We believe that the least we can do is to show that we can deliver reasonable results based on data acquired with a 7-year-old low frequency eye tracker and two very different methods of AOI production.

## Methods

### Scan path entropy

In the following recipe we present a method to compute scan path entropy. Before we start we make some choices. The method presented here aims at measuring gaze guidance to the brand logo (we could have made another choice here). We know that many ads are designed for other purposes than only fixating the brand logo as fast as possible; therefore we expect some of the ads in the present test to have little or no gaze guiding capacity to the brand logo. In the following recipe we compute the entropy from scan paths that end on the brand logo. This recipe can be applied to other AOIs than the brand logo and it can also be used without a target AOI. We will touch on this issue in the discussion.

1. Produce a set of AOIs. Keep in mind that smaller and more detailed AOIs result in both longer and a higher number of scan paths.
2. Choose a target AOI. In this example the target is the brand logo. This step may be skipped or another target may be chosen.
3. Transform each scan path into a character string. Each character refers to a fixation in a specific AOI. We will use “T” for target.
4. Remove all repetitions. “AAABCDDT” becomes “ABCDT”. In fact, this is going from a fixation-based representation to a dwell based representation (Holmqvist et al., 2011, page 190). A dwell consists of one or more fixation within one AOI.
5. Remove repetitions of two characters (“ABABCDT” becomes “ABCDT”). This is removing re-fixations from a pre-planned path. Re-fixations may occur if the previous fixation was too short to allow for visual analysis (Hooge and Erkelens, 1998; Hooge et al., 2007) and occur during search, reading and free viewing. We decided to remove these re-fixations because we believe they originate from timing errors in oculo-motor control, not from choosing an ineffective path.
6. Cut the character string after the first occurrence of T (“ABCDTEFG” becomes “ABCDT”).
7. Count the number of unique scan paths.
8. Construct a histogram of the unique scan paths and their frequency.
9. Apply the entropy formula to compute entropy.

### T_50_

To measure attention drawing power of an AOI one needs a measure that takes care of both the number of first AOI hits and the speed at which the attraction occurs. Such a measure is T_50_. T_50_ is extracted from the cumulative reaction time distribution.

**Figure 1:**
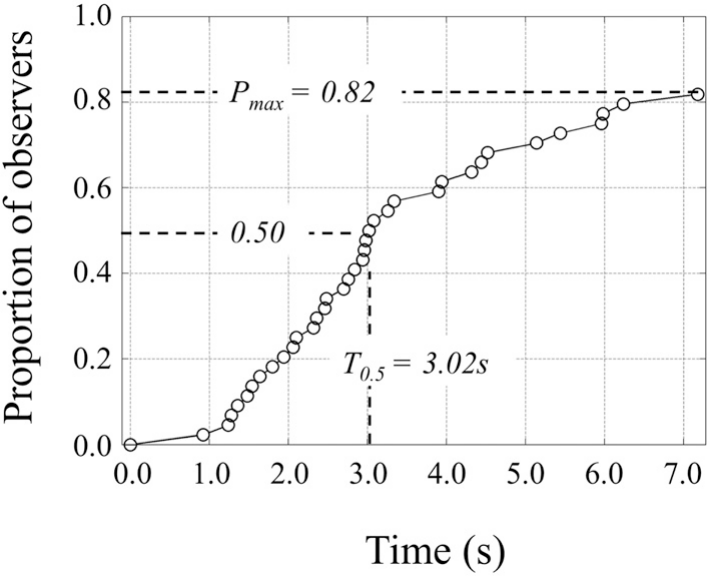
**Cumulative proportion of observers fixating a target AOI as function of time**. 50% of the observers fixated the target AOI in 3.02 s. The maximum proportion of observers fixating the target AOI was 0.82. We refer to this number as fixation score or *P*_max_.

1. Construct the cumulative reaction time distribution. Make a list of points of time of first AOI hits (Holmqvist et al., 2011, page 189). In this list there is a point of time for each observer fixating the AOI at least once. The number of points may be lower than the number of observers in the test, because some observers miss or skip the brand logo. If there is more than one AOI in the visual stimulus, make a “AOI first hit” list for each AOI.
2. Sort the list (ascending) and add a second column to put rank numbers. Transform the list to a relative distribution by dividing the rank number by the total number of observers that were exposed to this stimulus. The total number of observers may be larger that the number of observers that fixated the AOI (see point 1).
3. To explain T_50_ extraction, we made a figure having the sorted time on the x-axis and the relative rank on the y-axis (Figure 1). The time at which 50% of the observers fixated the target AOI (T_50_) can be determined graphically if sufficient observers fixated the target AOI. Alternatively T_40%_ or T_25%_ is a possible measure. Figure 1 shows T_50_ = 3.02 s. Of course graphical determination is not an option in a large experiment with many AOIs. If the number of observers is even, T_50_ can be determined directly from the list of relative rank numbers by taking the AOI first hit time of the N/2th observer or relative rank number 0.5. If the list does not contain a point with a relative rank number of 0.5, it can be determined by linear interpolation. The cumulative curves can also be fitted with a function (a Weibull function with 4 parameters is a suitable candidate).

(2) $$F\left(t;\alpha ,\beta ,\lambda ,\text{k}\right)=\alpha \left[1-e^{-{\left(\frac{\left(\text{t−}\beta \right)}{\lambda }\right)}^{k}}\right]$$

T_50_can then be determined by taking the inverse function and find the value for 0.5.

### Visualised transition matrices: arrow plot

**Figure 2:**
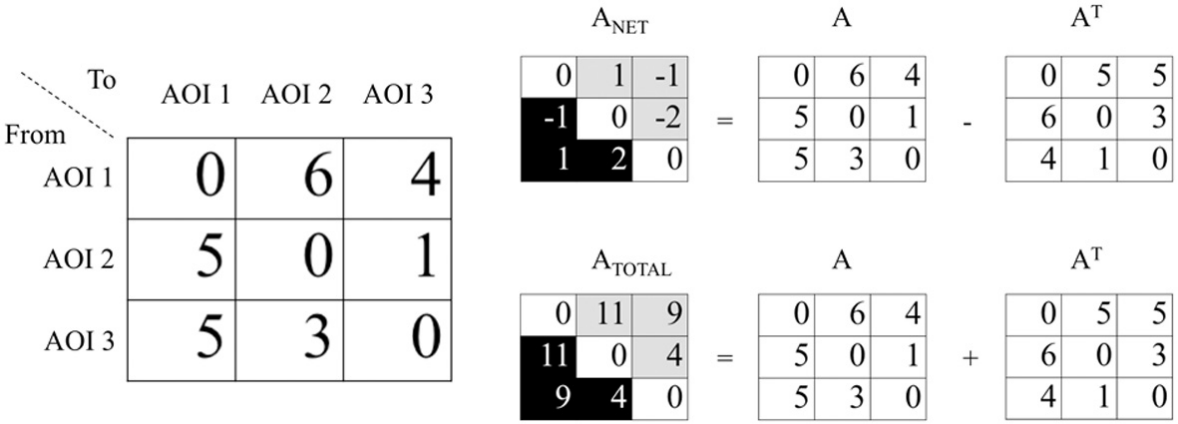
**Left panel**. Transition matrix: The diagonal contains zeros because by definition a transition is a saccade from one AOI to another. In this example there are 5 transitions from AOI nr 3 to AOI number 1. **Right panel**. The black cells in *A*_net_ describe the net transitions. The positive number in the third row, second column of *A*_net_ denotes 2 transitions from AOI nr 3 to AOI nr 2. The negative number in the second row, first column denotes one transition from AOI nr 1 to AOI nr 2 (negative number reverses the direction). The black cells in *A*_total_ describe the total number of transitions between three AOIs.

For the visualization of the eye movements we use the transition matrix. Transition matrix cells contain frequencies of direct transitions between AOIs. A transition is a saccade from one AOI to another one (Holmqvist et al., 2011, page 190/191). For this study we construct transition matrices with self-written matlab software, but there is also commercial software available for transition matrices production (BeGaze from SMI and Noldus observer that works with Tobii). To explain how we visualize transition matrices, we use an example with three AOIs (Figure 2). A transition matrix describing all transition between these three AOIs has nine cells (3 × 3). The cells on the diagonals are empty (a transition goes from one AOI to another). The cell in the first row and third column describes the number of transitions from AOI nr 1 to AOI nr 3. The cell in the third row and second column describes the number of transitions from AOI nr 3 to AOI nr 2. We decided to visualize transitions with two figures to avoid too much clutter in the resulting image. In one panel, we visualize the total number of transitions between AOIs with arrows with two arrowheads. In the other panel we visualize the net number of transitions between AOI. The net number of transitions has a direction, which will be indicated with an arrow with one arrowhead. For example, if there are 5 transitions from AOI nr 3 to AOI nr 1 and 4 transitions from AOI nr 1 to AOI nr 3, the net number of transitions from AOI nr 3 to AOI nr 1 is 1 (Figure 2). The net number of transitions between AOIs is calculated as follows

(3) $$A_{\text{net}}=A-A^{T}$$

With the transition matrix *A* and its mirror *A*^*T*^. The total number of transitions is

(4) $$A_{\text{total}}=A+A^{T}$$

Since we added and subtracted *A* and *A*^*T*^, the cells under and above the diagonal yield similar information. We are only interested in the number of transitions between each AOI pair and therefore we only need the numbers in the cells of the left bottom corner (Figure 2, the black cells in *A*_net_ and *A*_total_). If the observers make equal number of transitions between all the cells we expects no transitions in the net transition matrix. In contrast, we expect the net transition matrix obtained from saccades made in a stimulus with strong gaze guidance to yield many transitions. Therefore relative number of transitions in the net transition matrix is reported with the figure. This number is calculated as follows

(5) $$F=\frac{\sum A_{\text{net}}}{\sum A_{\text{total}}}$$

To produce transition figures with arrows between the AOIs, we transformed the numbers in both the total and the net transition matrix to relative numbers. The width of the arrows is scaled with the maximum relative number in the matrix.

## Experiment + methods

### Area of interest production

**Figure 3:**
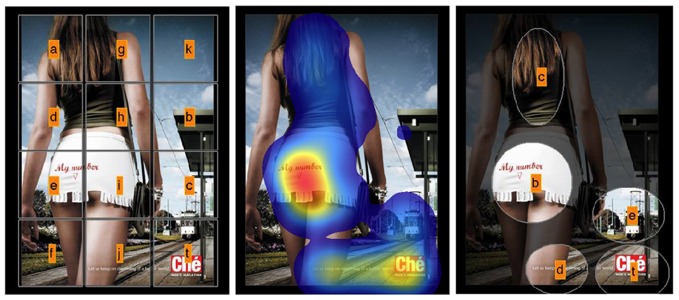
**Two methods for AOI production used in this study**. The **left panel** contain the gridded AOIs. The **right panel** contains the hand drawn AOIs. These AOIs are based on the heat map **(middle panel)** and aim at capturing fixation clusters. The target AOI (the brand logo) is coded with “*t*”. This manuscript is not about AOI-production. We used these two extreme methods to show the robustness of our measures, not to show any preference for one of the two methods.

Eye movements were analyzed with 2 different sets of AOIs. In the clustered condition, AOIs were hand drawn in Adobe Photoshop and based on the heat map. This is a data driven approach and all clusters of fixation received an AOI. In the gridded condition, the stimulus area was divided in 12 equal rectangles. Horizontally oriented displays were divided in a 3×4 grid and vertically oriented stimuli were divided in a 4×3 grid. Examples of clustered and gridded AOIs can be found in Figure 3.

### Observers and procedure

For this study we recorded the eye movements from of 54 observers (27 males, 27 females, age ranged from 18 to 55). Observers were positioned in front of a Tobii 1750 Eye Tracker and were individually calibrated with a nine-point calibration. They were instructed to browse the ads as they would have done when they would stumble upon them in a magazine (this instruction is comparable to that of Pieters and Wedel, 2004), by clicking left arrow button they could go on to the next ad. In between stimulus presentation a black screen was presented.

### Material

For the study we used unmodified advertisements downloaded from the Internet. The criteria for an ad to be included in the study were that it was available in a sufficiently high resolution, and that the language used in the ad was either Dutch or English or a mixture of the two. We choose 70 different digital ads that were all scaled down to the maximum size possible to be presented on the screen of the Tobii 1750 (1280 pixels horizontally, 1024 pixels vertically), and depending on the orientation of the original ad, the rest of the screen was filled by a black background. Not all lay-outs of the chosen ads were suitable for the analysis done in this study. We included 39 of the 70 ads in the analysis with both the gridded AOIs and the clustered AOIs. These 39 ads met the following criteria.

1. The ad contains one brand logo
2. The brand logo is smaller than an AOI of the gridded condition
3. The brand logo is located in one of the 12 gridded AOIs except the two center ones.

### Fixation detection

We performed fixation detection by a computer program that marked fixations by an adaptive velocity threshold method. First, velocities were obtained by fitting a parabola through three subsequent data points of the position signal. We used the derivative of this parabola to estimate the value of the velocity of the second (center) data point. This procedure was repeated for all data points (except the first and the last point). In the present analysis, everything that is not a saccade is called a fixation. To remove the saccades from the signal we calculated average and standard deviation from the absolute velocity signal (composed from the vertical and horizontal velocity signal). All data points having absolute velocities higher than the average velocity plus 3 times the standard deviation were removed. This procedure was repeated until the velocity threshold converged to a constant value or the number of repetitions reached 50. Then we removed fixations having durations shorter than 60 ms from the analysis. We removed saccades smaller than 1.0°. When a saccade was removed, the preceding and succeeding fixations were added together. This is a velocity based fixation detection method suitable for data from low frequency eye trackers such as Tobii 1750 (50 Hz), Tobii T60 (60 Hz), EasyGaze (52 Hz max) and SMI red (60–120Hz). One may avoid fixation detection by directly calculating the dwell time from raw data in combination with the AOIs at the cost of too long dwell times because parts of the saccade time are counted as dwell.

## Results

### Data quality and data exclusion

We measured data quality in each trial by determining RMS noise (Holmqvist et al., 2011 page 35) in the horizontal and vertical eye movement signal during fixation (meaning the saccades were removed from this signal) and saccade detection velocity threshold. RMS noise is a measure for the variable error and its cause may vary from physiology to operator quality (Holmqvist et al., 2012; Nyström et al., 2013). Average RMS noise level over all trials and observers measured 0.28° for the x-component and 0.33° for the y-component, average saccade detection velocity threshold converged to 24.5°/s. We combined horizontal and vertical RMS noise in one number by adding up the horizontal and vertical component using the theorem of Pythagoras. From these values we constructed per observer one histogram of RMS values (each trial delivers one value). From the original data set of 54 observers we removed 6 observers due to high values for mean combined (horizontal and vertical) RMS noise (>30 pixels/±0.8°).

### T_50_

**Figure 4:**
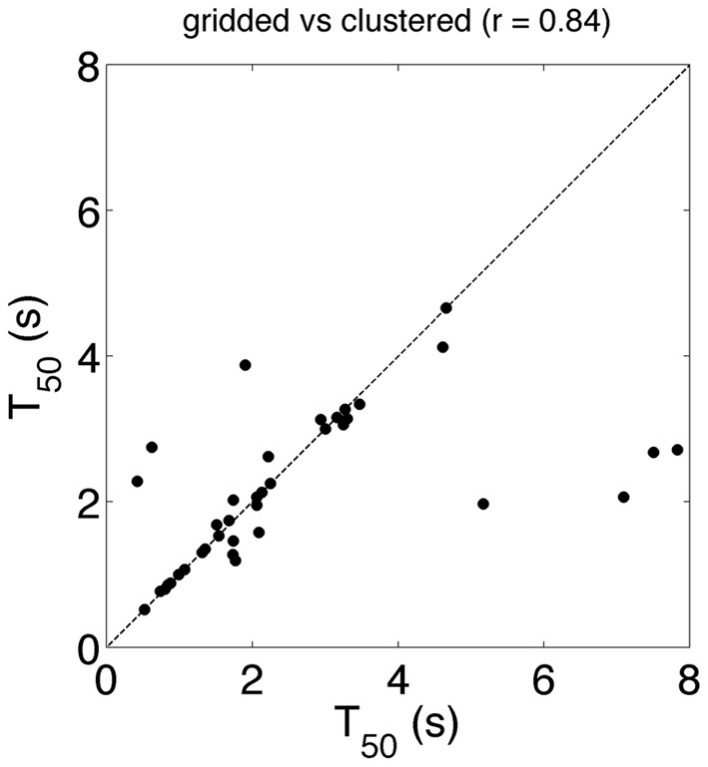
**T_50_determined from gridded AOI vs. T_50_ determined from clustered AOIs**. Each data point represents data from 48 observers in one ad. T_50_ represents the time that is required for an AOI to attract fixations from the first unique 50% observers of the population.

To estimate attention drawing power of brand logos we determined T_50_ of the brand logo in 39 different ads. To rule out the possibility that our results are due to the choice of AOIs we choose to make our AOIs in two very different ways. We refer to the two methods as gridded and clustered AOIs. Figure 4 shows values for T_50_. Each dot in the figure represents data for one advertisement; the *x*-value is T_50_ obtained from the clustered AOIs and the *y*-value is the T_50_ obtained from the gridded AOIs. T_50_ ranges from 0.42 to 7.8 s for the clustered AOIs and from 0.52 to 4.66 s for the gridded AOIs. T_50_ reaction times are shorter for gridded AOIs than for clustered AOIs. This is probably due to the larger size of gridded AOIs. We find a high correlation *r* = 0.84 for T_50_ determined between two very different methods.

### Brand logo fixation

**Figure 5:**
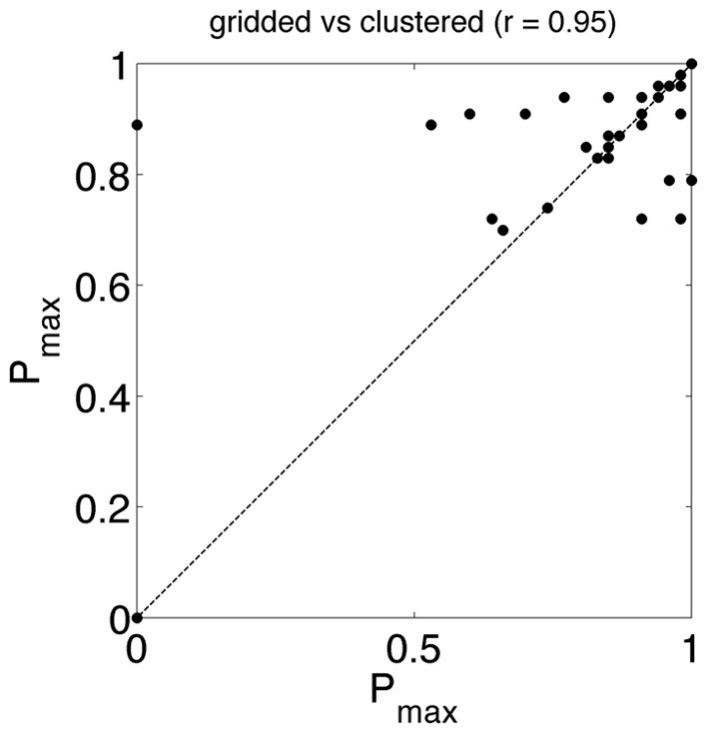
***P*_max_ (see Figure 1) obtained from gridded AOIs vs. *P*_max_ obtained from clustered AOIs**. *P*_max_ refers to the proportion observers that fixated a target AOI (in this study the AOI with the brand logo) at least one time. In most ads the *P*_max_ is high (>0.8) and the correlation between the *P*_max_ from gridded vs. clustered is high.

Analogous to accuracy in a visual search task we determine *P*_max_ or fixation score in this free viewing task (Figure 1, *P*_max_ = 0.8 means that 80% of the observers fixated the brand logo at least once). The fixation score of the brand logo AOI is high, irrespective of AOI production method. Most ads have a fixation score higher than 0.8 and the correlation between the scores is 0.95 (see Figure 5).

### Entropy

**Figure 6:**
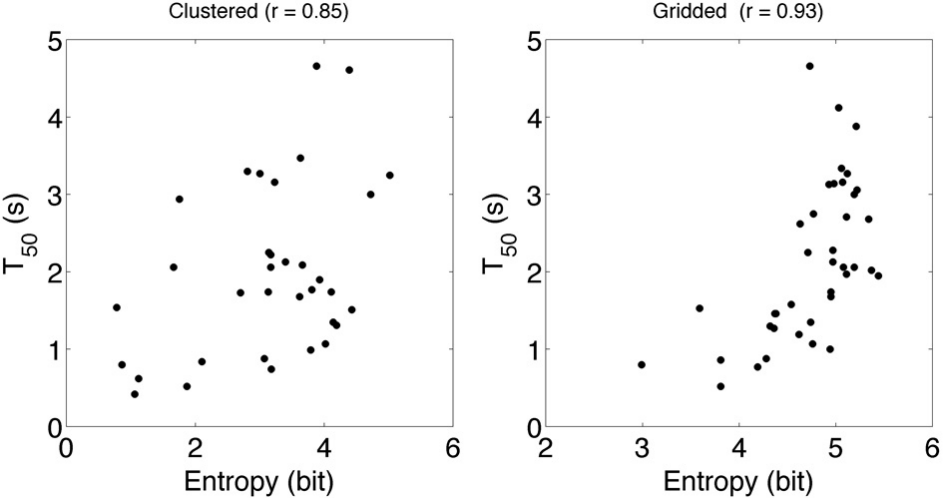
**T_50_ vs. Entropy determined from gridded AOIs (left panel) vs. T_50_ vs. Entropy determined from clustered AOIs**. Each data point represents data from 48 observers in one ad. Both T_50_ and entropy values differ for the 2 AOI production methods. However, in both cases T_50_ and Entropy have a high correlation.

We stated before that if the scan path entropy measure is sensitive, it may be a useful measure. Entropy obtained from the clustered AOIs ranges from 0.78 to 5.11 and entropy from the gridded AOIs ranges from 2.99 to 5.44. We stated that we expect a correlation between T_50_ and scan path entropy. Figure 6 shows T_50_ vs. scan path entropy.

**Figure 7:**
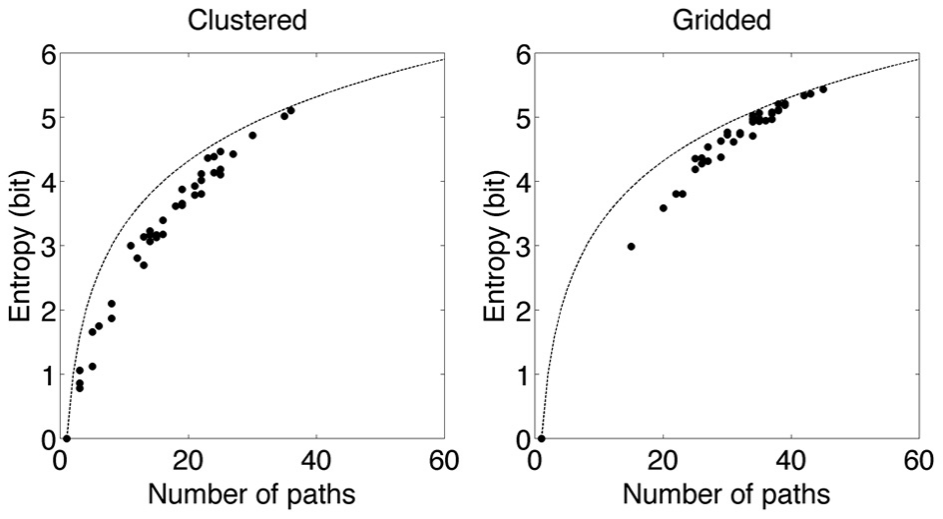
**Entropy as function of the number of scan paths**. A single dot represents data from an individual advertisement. The line denotes the maximum entropy given the number of paths. The previous is calculated from a flat distribution of scan paths. In the gridded condition for high number of paths the measured entropy resembles the maximum entropy for that specific number of paths. In the clustered condition for both high and low number of paths the measured entropy resembles the maximum entropy for that specific number of paths.

Is the scan path entropy high or low? The best way to answer that question is to compare the scan path entropy to the minimum and maximum entropy possible. Minimal entropy would be reached if all subjects follow the same scan path. If this is not the case the number of scan paths is always higher than one. To determine the maximum entropy is difficult. Here we compared obtained entropy with the maximum entropy for the same number of paths (Figure 7 dashed line). For the highest number of paths, entropy almost resembles the maximum entropy (for both AOI production methods). For the lower number of paths, entropy is lower than the maximum entropy for the number of paths (gridded AOIs). With clustered AOIs, for the lowest number of paths entropy almost resembles the maximum entropy. An explanation is not complicated. Ads without gaze guidance produce many different paths without preference for any the paths. This results in maximal entropy. Ads with gaze guidance produce fewer paths than possible and some of these path were favored resulting in lower entropy. For the low number of paths (around 1, and 3) the difference between minimal and maximal is quite small.

### Visualization

**Figure 8:**
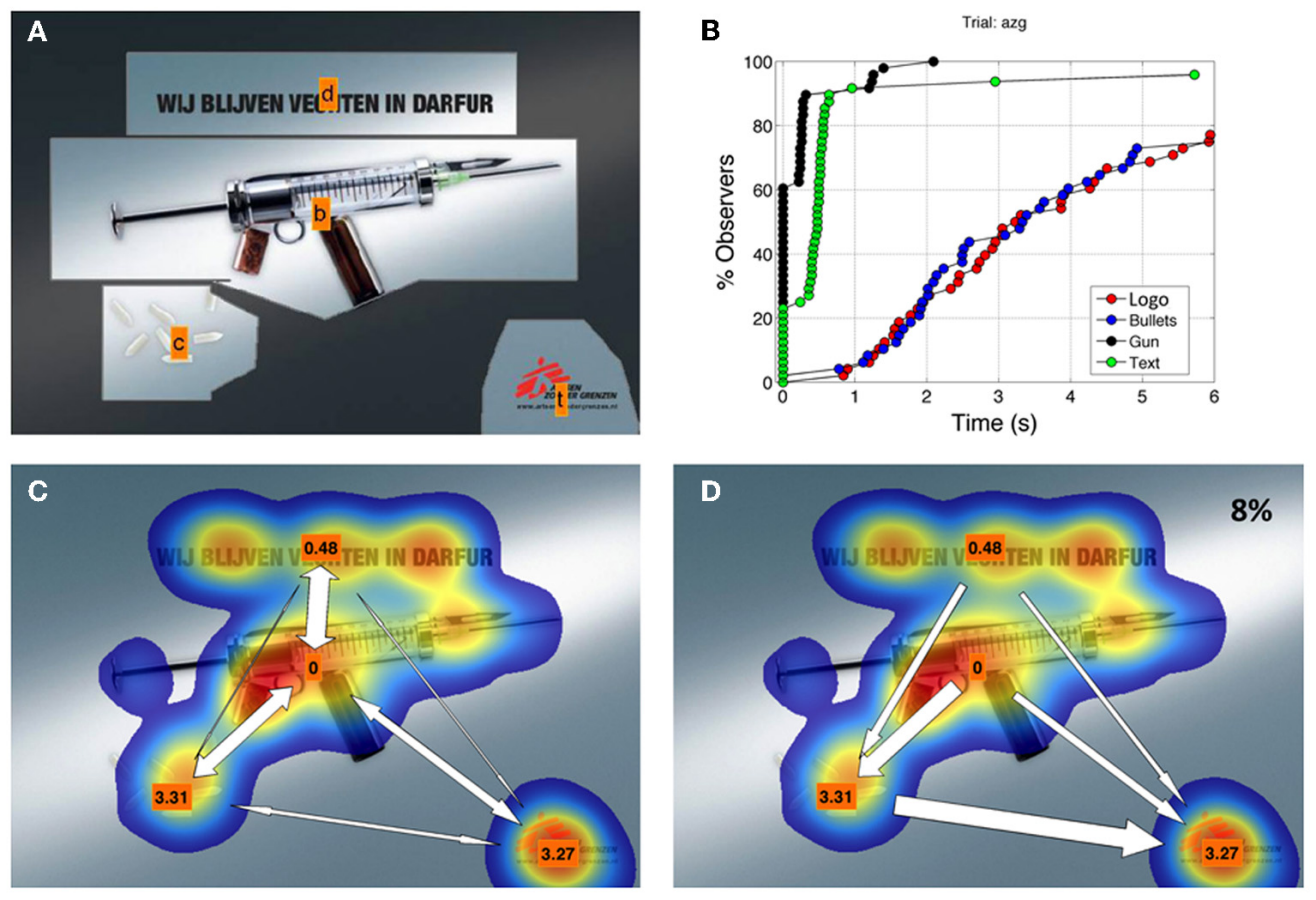
Panel **(A)** Hand drawn AOIs. The shape and size of the AOI are not very critical in sparse displays. Panel **(B)** Cumulative plots for 4 AOIs of panel **(A)**. Cumulative curves for AOI-Text and AOI-Gun are steep with high fixation scores (100 and 98%) and short T_50_ (0s and 0.48 s). Panel **(C)** shows visualization of the total transition matrix. The orange labels denote T_50._ The gun is fixated first and many transitions go to the text. Later there are transitions to the bullets (left) and the brand logo (right). Panel **(D)**. shows a visualization of the net transition matrix. Only 8% of the transitions are in this figure, indicating that gaze guidance is not very strong in this stimulus.

**Figure 9:**
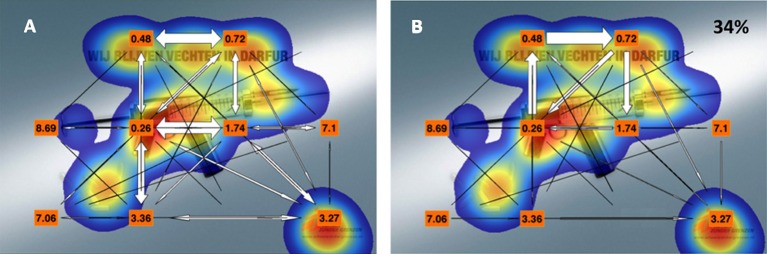
Panel **(A)** shows the total transition matrix. Orange labels denote T_50_. Panel **(B)** shows the net transition matrix. Panel **(B)** contains 34% of the transitions.

Figures 8 and 9 show two examples of scan path visualization. Figure 8 shows an example with a low number of AOIs. It was already clear in the visualizations of Lessing and Linge (2002) that too many elements cause clutter and thus make the figure less usable as a visual interface. One way to avoid clutter is splitting up the visualization in two panels, namely the total and the net transitions. We scale the width of the arrows to code for the relative number of transitions (no numbers required, again less clutter). Figure 8C shows an arrow plot depicting the total relative number of transitions. It also yields T_50_ values. From this figure we can see that scanning started in the center. Figure 8C shows many transitions between the text and the visual (the gun). Figure 8D shows the net transition and it contains only 8% of the transitions. This is an indication for low gaze guidance capacity. With this knowledge we can look to panel **(B)**, showing T_50_ for all AOIs. Here we can see that after quickly fixating both the gun and the text AOIs, it takes a long time before the observers gaze at the bullets and the brand logo. The T_50_ for the bullets (3.31 s) and the brand logo (3.27 s) suggest that these two AOIs compete for attention. If we had to advice the designer we would ask to make the bullets a less attractive target or make the brand logo stand out better, facilitating quicker brand logo fixation. A re-test could be used to validate the changes applied to the design. Figure 9 clearly shows the limitation of our visualization method. Too many AOIs make the figure hard to interpret. However, a global scan pattern can still be extracted from this figure. Especially in the top of Figure 9C (the net transitions), reading from left to right is clearly visible.

## Discussion

### Designing a test

What to measure and how to test eye movement behavior with the new quantitative measures? In case of ad enhancement we suggest to build a database from quantitative eye tracking measures (including T_50_, entropy and (visualized) transition matrices). New ads should be measured each week with a large number of observers (±50–100). However, such an approach is too expensive for most usability and marketing research companies. Interpreting quantitative measures is also possible based on a simple differential measurement instead of a large database. This is of course much cheaper and easier to carry out.

Imagine a client that provides a researcher with an ad and the question whether the design is good enough (it may sound strange, but this is usual practice). The researcher should be provided with (slightly) different versions of the ad, to be able to carry out a differential measurement to allow for interpreting the quantitative eye tracking measures. Then T_50_ and entropy could be used to choose the best design. In addition, if there is the possibility to interview the designer or client, the researcher should ask for design goals, hypothesis, discussions about and ideas behind the design. Comparing ideas and goals of designers with actual saccadic scanning behavior may help improving the design and evoke new ideas; the arrow plots can be helpful in this process. For example, there is still strong belief in the field that observers scan ads in a Z-pattern. The idea behind Z-scanning is that people scan from left to right and from top to bottom (in a reading like way). In this study we saw many different scan-patterns (including z-scanning, but of course not exclusively Z-scanning). As stated before when different versions of the ad compete for publication the shortest brand logo T_50_ may be used as criterion. However, depending on the goal of the ad, a mix of T_50_ values (for attention drawing capacity) combined with total dwell times (for attention retaining capacity) would do the job of choosing the best ad. We restricted ourselves in this study to T_50_ measures for the brand logo for reasons of simplicity.

If the designer provides only one ad, the researcher could add ads from competing companies or ads having similar composition to the eye tracking test. Doing research without direct competitors from the same designer or company is of course more difficult and less effective. One can always give qualitative description of scan patterns as in Figure 8. Differential measurement within one ad is also possible. An example of this is provided in results section about *Visualization*.

### Doing statistics with T_50_ and entropy

In the present article we did not yet use statistics. Imagine that there is doubt about the sensitivity of T_50_ or scan path entropy in a situation where two visual stimuli produce almost similar values. To solve this problem, we advise to use a bootstrapping method (Efron, 1979). Bootstrapping is a resampling method that uses an estimator (such as T_50_) based on a subpopulation of responses drawn from the whole population. In bootstrapping, drawing a subpopulation is repeated a large number of times (in the range of 5000–10,000 times).

### Relation between choice of aois, T_50_ and entropy

How sensitive are T_50_ and scan path entropy for the choice of AOI? In this study we used two completely different methods to produce AOIs and we found correlated but different values for T_50_ and scan path entropy (Figures 4, 6 and 7). It may depend on the nature of the visual stimulus how T_50_ and scan path entropy are related to the AOIs. If the visual stimulus is sparse, it is recommended to make the AOIs as large as possible. That is possible because in such stimuli there is not much crowding and conspicuity areas of visual elements are large (Engel, 1971; Toet and Levi, 1992). Large conspicuity areas implicate that objects are visible at larger eccentricities (or larger distance from the gaze point), allowing observers to overview larger areas around the gaze point.

In dense stimuli, however, researchers may make many choices during production of AOIs that can affect AOI measures. An example can be found in Figure 8C, where two fixation clusters can be found in the gun AOI. The previous makes clear that comparison of AOI measures (such as T_50_, scan path entropy and total dwell time) between different studies may be impossible without taking into account the nature of the AOIs. Another problem is that in dense visual stimuli the number of AOIs may become too large to produce both entropy values that are interpretable and arrow plots that are informative. Here are some practical suggestions

1. Compute entropy from scan paths produced in the first second
2. Compute entropy for the first 5 dwells
3. Only select dwells from large clusters
4. Produce large AOIs
5. Investigate entropy in one section (upper left corner)
6. Remove fixation clusters that do not contain a minimal number of fixations

Applying one of the points above may be helpful in doing effective research, however any of the choices should be reported and motivated.

### Limitations of the arrow plot

The arrow plot is beautiful and appealing but Figure 9 shows clearly that if the number of AOIs exceeds a certain number (4 or 5), the arrow plot becomes cluttered and hard to interpret. Both restrictions to the data set and some modifications to the arrow plot would be helpful to avoid too much cluttering and too many arrows.

1. Only visualize transitions made in the first second of stimulus presentation
2. Only visualize the first n transitions
3. Remove arrows that contain less than *p* % of the data (for example *p* = 1)
4. Only visualize transitions made before target AOI fixation (in contrast to this, in Figures 8 and 9 we included all data)

There are other modifications possible to the arrow plot. The width of the arrows code relative numbers of transitions in the present figures. The width of the arrows in the net and total transition arrow plot is determined independently. They can be coupled together or made to represent absolute numbers of transitions. Another possibility is to measure eye movements in two different visual stimuli and color-code the arrows if they represent a significantly higher number of transitions in one of the two visual stimuli.

### Entropy is a useful measure in practical situations

Why use entropy as a measure to qualify ads? There is a correlation between scan path entropy and T_50_ but T_50_ is a direct measure and scan path entropy is an indirect one. If one is only interested in direct performance measures as total dwell time and T_50_, one should not determine entropy. However, if advertisement enhancement is of interest, both entropy (or the underlying histogram from which entropy is calculated) and the arrow plot may be handy tools. They may provide insight in the cause of scan patterns that produce long or short T_50_s. Consider the following: The attention attracting power of brand logos can be increased easily by

1. increasing their size,
2. creating empty space around them or
3. giving them more luminance contrast

to make them more conspicuous (Toet and Levi, 1992; Kooi et al., 1994). However, designers may have many reasons not to increase conspicuity for aesthetic reasons or because they have stick to corporate standard design. In that case increasing gaze guidance capacity is an alternative strategy to make observers look to a specific element.

There is another reason to be interested in scan path entropy. Many advertisements contain a story that is important for message transfer. It is assumed that fixation order is important for the observer to understand the story (whether that is really the case is an interesting question too). Scan path histograms, scan path entropy and arrow plots may provide the necessary information to investigate this.

## Conclusion

We suggest a new visualization method “the arrow plot” in combination with two quantitative measures, T_50_ and scan path entropy. These methods were applied on 39 ads and we showed with two methods of AOI production that T_50_ and scan path entropy are robust measures. The arrow plot reveals aspects of average scanning behavior that are hidden with attention maps. We discussed the pros and cons and suggested ways to adapt the new measures and visualizations to specific research questions. Our new methods will be applicable to the field of art and eye movements, the field of psychology (free-viewing and visual search) and the fields of ergonomics and usability.

### Conflict of interest statement

The authors declare that the research was conducted in the absence of any commercial or financial relationships that could be construed as a potential conflict of interest.
